# Auscultation in Uterine Hemorrhage

**Published:** 1879-11

**Authors:** 


AUSCULTATION IN UTERINE HEMORRHAGE.

Prof. Depaul in a clinical lecture (*Gaz. des Hosp.*) observes that when hemorrhage occurs during labor, it will generally be found to arise from partial detachment of the placenta, the cord being too short. "I remember," he said, "the case of a young woman whose delivery had gone on very well, when, as the head was approaching the vulva, two or three spoonfuls of blood suddenly appeared between the thighs. I immediately practiced auscultation, and found the fœtal heart beating irregularly. It was evident that the infant was suffering, and that it was dangerous to await the natural termination of the labor which might last two or three hours longer. Dilatation was complete. Easily persuading the mother of the necessity of terminating the labor rapidly, I applied the forceps. Immediately after the child was extracted there followed five or six enormous clots, weighing about a couple of pounds. The child was born respiring with difficulty, but soon quite recovered. Never forget that whenever you meet with a flow of blood, to assure yourself by auscultation as to the state of the infant, and when dilatation has taken place, hasten to interfere whenever life seems in danger."—*Medical Times and Gazette.*

